# Supplementary material for: Time‑resolved multi-omic analysis of paclitaxel exposure in human iPSC‑derived sensory neurons unveils mechanisms of chemotherapy‑induced peripheral neuropathy
Source: Cell Death Dis. 2026 Feb 10;17(1):211. doi: 10.1038/s41419-026-08445-2 (PMC12921266; doi:10.1038/s41419-026-08445-2)
Supplement: Supplementary file 15 — Dataset 1. [file 41419_2026_8445_MOESM15_ESM.zip › CDD_Open_Data/05_Protein/03_Evidence_Plots_Andranik/GOBP_POSITIVE_REGULATION_OF_JNK_CASCADE.pdf]

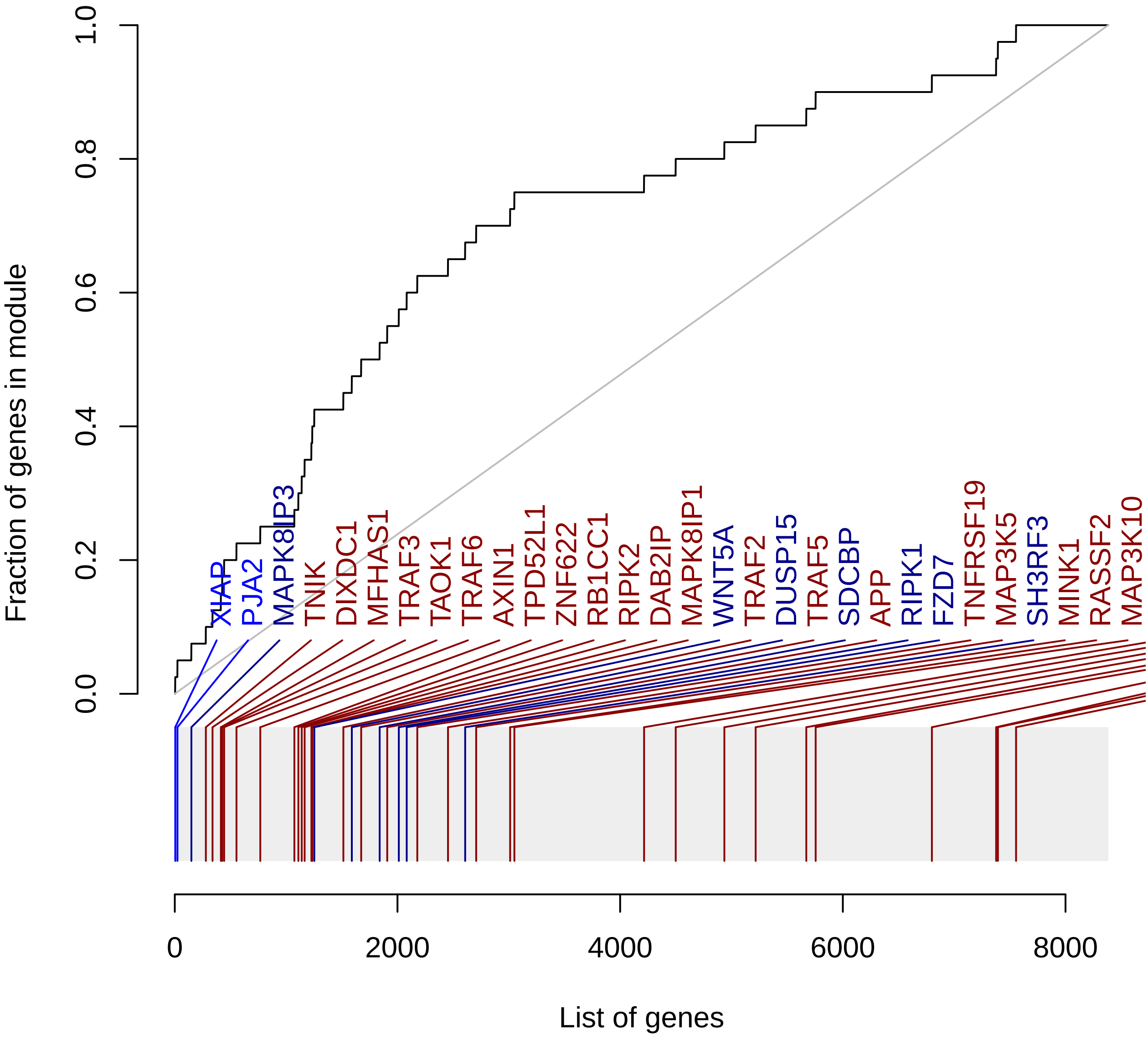

Fraction of genes in module
0.0
0.2
0.4
0.6
0.8
1.0
XIAP
PJA2
MAPK8IP3
TNIK
DIXDC1
MFHAS1
TRAF3
TAOK1
TRAF6
AXIN1
TPD52L1
ZNF622
RB1CC1
RIPK2
DAB2IP
MAPK8IP1
WNT5A
TRAF2
DUSP15
TRAF5
SDCBP
APP
RIPK1
FZD7
TNFRSF19
MAP3K5
SH3RF3
MINK1
RASSF2
MAP3K10
0
2000
4000
6000
8000
List of genes
